# The effect of exposure to farmed salmon on piscine orthoreovirus infection and fitness in wild Pacific salmon in British Columbia, Canada

**DOI:** 10.1371/journal.pone.0188793

**Published:** 2017-12-13

**Authors:** Alexandra Morton, Richard Routledge, Stacey Hrushowy, Molly Kibenge, Frederick Kibenge

**Affiliations:** 1 Raincoast Research Society, Sointula, British Columbia, Canada; 2 Department of Statistics and Actuarial Science, Simon Fraser University, Burnaby, British Columbia, Canada; 3 Department of Biological Sciences, Simon Fraser University, Burnaby, British Columbia, Canada; 4 Department of Pathology and Microbiology, Atlantic Veterinary College, University of Prince Edward Island, Charlottetown, Prince Edward Island, Canada; Friedrich-Loeffler-Institut Bundesforschungsinstitut fur Tiergesundheit, GERMANY

## Abstract

The disease Heart and Skeletal Muscle Inflammation (HSMI) is causing substantial economic losses to the Norwegian salmon farming industry where the causative agent, piscine orthoreovirus (PRV), is reportedly spreading from farmed to wild Atlantic salmon (*Salmo salar*) with as yet undetermined impacts. To assess if PRV infection is epidemiologically linked between wild and farmed salmon in the eastern Pacific, wild Pacific salmon (*Oncorhynchus* sp.) from regions designated as high or low exposure to salmon farms and farmed Atlantic salmon reared in British Columbia (BC) were tested for PRV. The proportion of PRV infection in wild fish was related to exposure to salmon farms (*p* = 0.0097). PRV was detected in: 95% of farmed Atlantic salmon, 37–45% of wild salmon from regions highly exposed to salmon farms and 5% of wild salmon from the regions furthest from salmon farms. The proportion of PRV infection was also significantly lower (*p* = 0.0008) where wild salmon had been challenged by an arduous return migration into high-elevation spawning habitat. Inter-annual PRV infection declined in both wild and farmed salmon from 2012–2013 (*p* ≤ 0.002). These results suggest that PRV transfer is occurring from farmed Atlantic salmon to wild Pacific salmon, that infection in farmed salmon may be influencing infection rates in wild salmon, and that this may pose a risk of reduced fitness in wild salmon impacting their survival and reproduction.

## Introduction

Infectious viruses are imposing a significant impact on the global salmon farming industry [1], where high host density can elevate both pathogen production and virulence above levels generally found in wild salmon [2]. Reduction in wild salmon productivity has been related to the scale [3] and presence [4] of salmon farms, and pathogen surveillance provides useful insight into declining wild salmon populations [5]. Nonetheless, few studies have examined the relationship between exposure to salmon farms and the proportion of wild salmon infected with specific viruses [6].

The piscine orthoreovirus (PRV), discovered in 2010 [7], belongs to the family *Reoviridae*, subfamily *Spinareovirinae* [8], and is now considered ubiquitous in marine farmed Atlantic salmon (*Salmo salar*) in Norway and British Columbia (BC), Canada [9, 10]. PRV is the causative agent of the disease heart and skeletal muscle inflammation (HSMI) [11], which causes specific lesions in the heart and skeletal muscle and can result in anorexia and abnormal swimming behavior in affected fish [9, 10, 11]. An HSMI outbreak can cause 100% morbidity in a salmon farm [11, 12] with associated mortality between 0 and 20% [12]. Stressors, such as sea lice treatment, bacterial infection, and algae blooms, appear to trigger the development of HSMI in PRV-infected fish [13, 14].

PRV infection is also associated with melanized foci in white muscle in Atlantic salmon in Norway [15]. A PRV variant (genotype II) is associated with HSMI-like disease in farmed coho salmon (*Onchorhynchus kisutch*) in Chile [16] and rainbow trout (*O*. *mykiss*) in Norway [17, 18]. Recently, another related orthoreovirus (PRV-2) was demonstrated as the etiologic agent of erythrocytic inclusion body syndrome (EIBS), a condition associated with mass mortality in farmed juvenile coho salmon in Japan [19]. PRV sequences have also been detected in rainbow trout in Chile that were affected by idiopathic syndrome of rainbow trout (ISRT) [20], and another potential member of the PRV group was associated with epidemic mortality in wild largemouth bass (*Micropterus salmoides*) in the USA [21]. PRV and related orthoreoviruses of fish are therefore not only of major economic concern to the salmon aquaculture industry worldwide, but also with significant consequences for conservation and fisheries of wild salmon.

Recent virus challenge studies with Atlantic salmon show that initially PRV causes a transient acute infection of the erythrocytes (red blood cells), which are nucleated in fish, where it replicates rapidly infecting up to 50% of the red blood cell population [9, 22, 23, 24]. PRV becomes detectable in other organs subsequent to this initial blood-borne infection [11, 22, 23]. HSMI is not detectable within the first 8–10 weeks post challenge [25].

The science on the infection dynamics of PRV in wild fish populations is still emerging. Garseth et al. [5] provide molecular-based evidence that salmon farms play a significant role in the long-distance transport and transmission of PRV in Norway, speculating that pathogen exchange solely between wild salmon during the at-sea migration phase likely plays a minor role in PRV dispersal. While PRV infection in Norwegian sea trout (*Salmo trutta*) is low (1.9–3.0%), the species’ persistence in the nearshore environment elevates exposure to salmon aquaculture. This heightens the possibility that sea trout could serve as an intermediary host for aquaculture-source PRV through habitat overlap with salmon during the freshwater spawning and juvenile rearing phases [26]. While no evidence of HSMI was detected in Norwegian wild salmonids [26], the researchers postulated that the impact of severe heart and skeletal muscle damage on a salmon’s cardiovascular capacity could decrease the likelihood of an infected fish entering the riverine habitat where sampling was conducted. It is widely observed that diseased wild fish are typically difficult to sample because they are preferentially removed from the population by predators [27].

**Fig 1:**
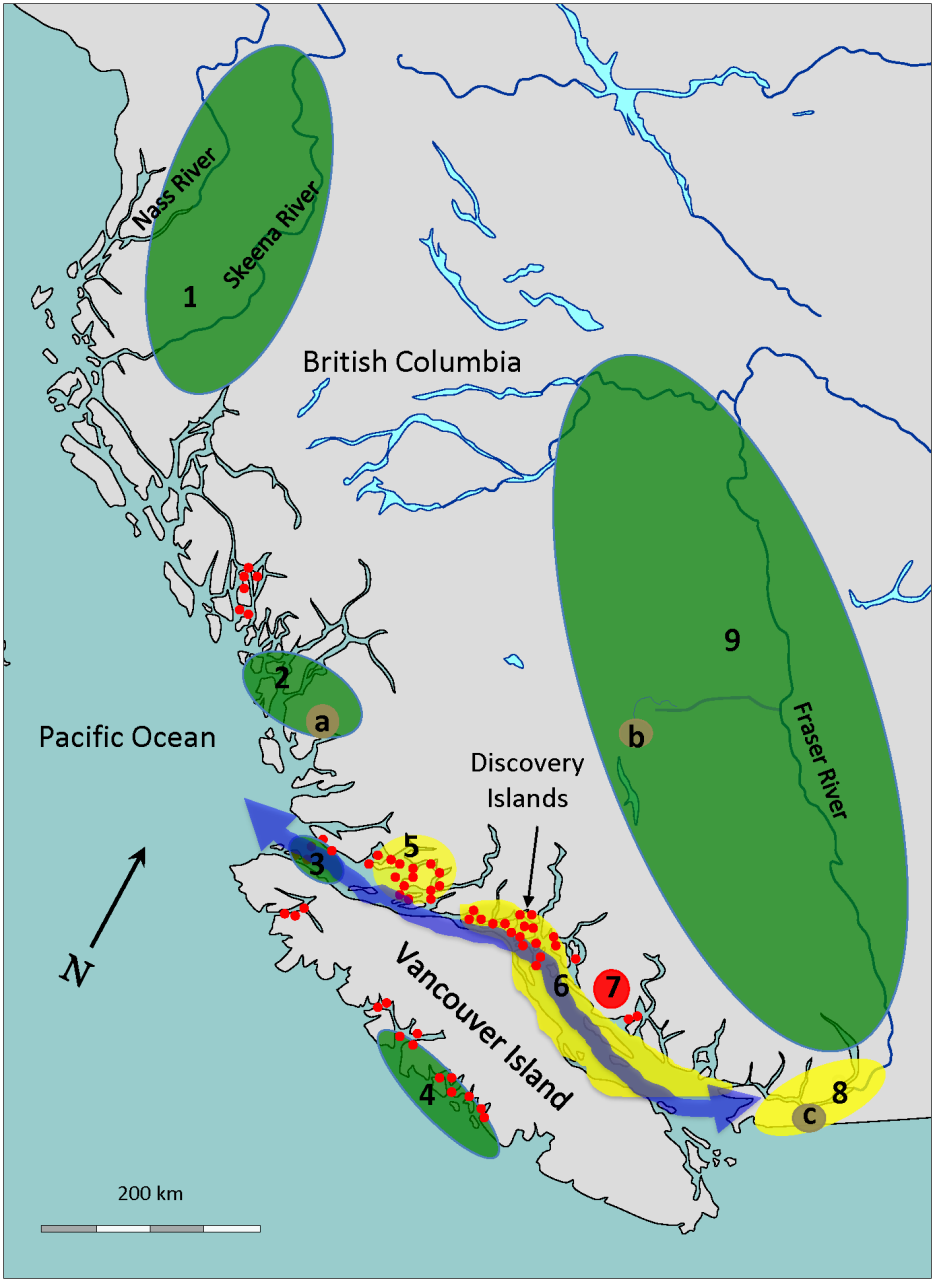
Map of BC with salmon farms and regions sampled. This map shows the following: (1) locations of salmon farms (red dots), (2) the 9 regions where wild salmon were sampled, (3) three lakes discussed in the text, (a) Oweekeno (elevation 15 m) (b) Chilko (elevation 1172 m) and (c) Cultus (elevation 47 m), and three river systems also discussed, the Fraser, Skeena, and Nass. Region color corresponds to the cluster analysis in Fig 4. The blue arrow represents the major Fraser River salmon migration route [38].

Most BC marine salmon farms, which are distributed in clusters along the southern half of the BC coast (Fig 1), raise Atlantic salmon, while steelhead (*O*. *mykiss*) are farmed in BC lakes. Although the Atlantic salmon eggs that entered BC may not have come directly from Norway [28], the dominant strain of Atlantic salmon farmed in BC is the Norwegian ‘Mowi’ strain [29].

The BC Ministry of Agriculture has reported that approximately 80% of BC farmed Atlantic salmon are infected with PRV [30]). Previous research on PRV infection in wild salmon in BC includes failure to detect the virus in 200 wild salmon collected in 2008 [31], but also a later report that the virus has been common in BC farmed and wild salmon at statistically similar rates of infection since 1987 [32]. Siah et al. [33] also reported a well-established PRV presence in wild and farmed salmon from Alaska through BC to Washington State.

Experimental challenge studies show that PRV will transmit readily from Atlantic salmon to conspecifics through cohabitation [9, 22, 23, 34], as well as sockeye (*O*. *nerka*) and chinook salmon (*O*. *tshawytscha*). These infections result in high viral loads in the erythrocytes and kidney [9, 22, 23, 24, 34, 35]. However, evidence of immune activation in response to PRV infection is mixed. Dahle et al. [34] and Haatveit et al. [9] find that PRV infection strongly induces a wide number of interferon-regulated antiviral and MHC class I genes in Atlantic salmon red blood cells. Garver et al. [23] report only a modest antiviral immune response in Atlantic salmon red blood cells, and they fail to find this response in head kidney tissues. Similarly, Polinski et al. [24], find no upregulated innate immune gene expression in sockeye salmon head kidney tissues. The cause for this discrepancy is unclear. However, Dahle et al. [34] and Haatveit et al. [9] challenged with PRV-infected tissues from a field outbreak of HSMI in Norway, while Garver et al. [23] and Polinski et al. [24] performed their studies with a strain of PRV from BC. The samples used in the present study have yielded 14 PRV isolates [16, 36]. The discrepancies between published findings as they relate to the induction of immune responses in host salmon have not been resolved.

While Garver et al. [23] reported that western North American PRV fails to cause HSMI, Di Cicco et al. [13] reported on two HSMI outbreaks in a salmon farm in BC. Hence, while earlier work reported that HSMI does not occur in BC [23, 32, 33], it is now understood that HSMI does occur in BC. However, HSMI has not been reported in wild or captive Pacific salmon.

Here, we report the results of PRV screening of a broad collection of wild salmonids sampled throughout much of BC in 2012 and 2013, and samples of farmed Atlantic salmon and steelhead reared in BC net pen facilities from the same time period. We assess these data for evidence of (i) a potential epidemiological link between farmed and wild salmon and (ii) potential impact of PRV infection on wild fish. In addition, we also present data, sampled from Oweekeno Lake between 2014 and 2016, on PRV infection status of wild salmonids including an endangered sockeye salmon population (S1 and S2 Tables).

## Materials and methods

### Sampling

As per restricted direct access to farm-specific Atlantic salmon, samples were obtained from markets selling fresh farmed salmon reared in BC marine net pen facilities. In 2012–2013, gill and head kidney samples were collected from 262 fresh farmed BC Atlantic salmon and 35 farmed Steelhead reared in freshwater net pens purchased from 10 BC market chains located in southwestern BC on 93 different dates. The fish suppliers confirmed that these farm salmon had been reared in the pens sited on the BC coast. There was no information as to the specific farm each sample was from. The “Best Before” date was used to select for the freshest samples.

In 2012–2013, gill, heart, head kidney, and spleen tissues were extracted from 601 wild Pacific salmonids (*Oncorhynchus* spp.) (Table 1) collected from marine and freshwater throughout southern British Columbia from the numbered Regions in Fig 1. Another 402 salmonids were sampled 2014–2016 from Oweekeno Lake, Region 2a (Fig 1, S1 Table). Because these were sampled during different years, they were analyzed separately.

**Table 1:** Numbers of wild salmon and trout collected in 2012 and 2013 by species and life stage. Numbers inside brackets are for the subset “exposed” to salmon farms, i.e. from Regions 5, 6, 7, 8 and 9.

Species	Juveniles	Adults	*Totals*
Chinook (*O*. *tshawytscha*)	22 (22)	77 (13)	*99 (35)*
Chum (*O*. *keta*)	23 (15)	44 (2)	*67 (17)*
Pink (*O*. *gorbuscha*)	32 (28)	76 (22)	*108 (50)*
Sockeye (*O*. *nerka*)	91 (3)	129 (74)	*220 (77)*
Coho (*O*. *kisutch*)	24 (23)	45 (8)	*69 (31)*
Steelhead (*O*. *mykiss*)	0 (0)	14 (9)	*14 (9)*
Kokanee (*O*. *kisutch*)	0 (0)	8 (1)	*8 (1)*
Trout (*O*. *mykiss/clarkii*)	0 (0)	16 (12)	*16 (12)*
**Totals**	**192 (91)**	**410 (141)**	***601 (232)***

We note that the sampling did not constitute an extensive, structured surveillance of wild salmonids in BC. Hence, we have not attempted to construct precise estimates of PRV prevalences in wild salmon with tight confidence limits. Our study, aimed at exploring potential geographic patterns and generating epidemiological evidence providing provisional support for key hypotheses, was more akin to those reported in [5] and [37].

The 192 juvenile salmon sampled in 2012–2013 were obtained from weekly beach seines conducted to monitor the spring outmigration through the near shore marine environment in Regions 5 and 6. These were collected under Fisheries and Oceans Canada scientific collection permits. The juvenile salmon collected from Oweekeno Lake in 2014–2016 were obtained via fixed trap nets, purse seining, and surface trawling. Adult salmon collected in 2012–2013 were opportunistically collected from marine sport and commercial fisheries in Regions 3, 5 and 6, and were obtained as freshly dead specimens from rivers in Regions 1, 2, 3, 4, 5, 6, 8, and 9. The Kokanee (*O*. *nerka*) sampled 2012–2013 from Region 7 and the trout sampled in the same years from Regions 2a, 7 and 8c were obtained from sport fisheries. One to ten fish were taken from each sampling event. The adult fish sampled in Oweekeno in 2014–2016 were collected via angling and gillnetting under BC Provincial licenses and from aboriginal food fisheries.

Percussion to the head was used to euthanize live fish. Tissues were extracted within hours after specimens were obtained, whether live-caught, from fisheries or purchased from markets, using aseptic technique, including fresh, disinfected tools (a separate set for external vs. internal sample removal), and disposable work surfaces for each fish. Tissue samples were preserved in RNAlater^®^ and shipped on ice to the Atlantic Veterinary College laboratory. No accompanying information on specific site identification or exposure classification was provided to laboratory analysts in order to minimize any bias. Cross contamination between samples from fisheries is expected and was minimized by sampling between different boats. In the case of sport-caught fish only 1–2 fish were sampled per boat.

### Regions

While the regional source of the farmed salmon could only be identified as the southern half of BC where salmon farms are established, the wild salmonids were collected from nine distinct geographic regions across BC (Fig 1). These regions, shown in Fig 1, are grouped into two categories which differ with respect to exposure to Atlantic salmon farms.

Regions 1 and 2 are distant from salmon farms, while Regions 3 and 4, though closer to salmon farms, are directly flushed by open-ocean water. Collectively, Regions 1–4 were classified as experiencing low exposure to Atlantic salmon farms (369 fish).

Regions 5 and 6 are inshore archipelago environments with high fish farm density and retentive marine circulation [39]. Region 7 is a lake that is inaccessible to anadromous fish, where a steelhead farm is sited. Regions 8 and 9, divide the lower and upper Fraser River at the strong rapids in the Fraser Canyon. A large percentage of sockeye, the second most numerically abundant salmon species in the Fraser River system [40], migrate through Region 6 as they approach the river to spawn [41]. Salmon from Regions 5–9 were therefore classified as having a high exposure to farmed Atlantic salmon (233 fish).

### Migration challenge

Fish sampled from the upper reaches of substantial watersheds (the Fraser, Skeena, and Nass, Fig 1) were deemed to have overcome significant migration challenges. The two largest of these watersheds are the Fraser and Skeena. For the Fraser, the most significant restriction is at Hells Gate in the Fraser Canyon (elevation about 100 m, but with the majority of the samples above this restriction taken from elevations of over 300 m). The primary salmon rivers in the Skeena watershed are the Babine, with major restriction in the vicinity of the 1951 Babine Slide (elevation around 400 m) [42], and the Bulkley, with major restriction at Moricetown Canyon (elevation around 380 m). Fish sampled from above these restrictions, and from above 300 m in another tributary, were placed in the high-challenge category. All fish sampled from the Nass were obtained from the Meziadin Lake watershed above the rapids in the Nass River, and were therefore also placed in this category.

### Viral screening

The laboratory was provided with a unique identification code for each sample which did not include information on the site or exposure classification. When the laboratory returned the results for the statistical analysis, the identification codes were used to link viral status to sampling location, species, and life stage.

RNA was manually extracted from fish tissues and quality was based on the OD A260/A280 ratio and quantitative reverse transcription polymerase chain reaction (RT-qPCR) amplification of either Atlantic salmon ELF-1α (GenBank accession number BT072490) or chinook salmon ELF-1α (GenBank accession number FJ890356) as an internal control. RNA was considered suitable for viral testing if amplification of ELF-1α yielded cycle threshold (Ct) values <30. Primers, probes, and RT-qPCR thermal cycling parameters were as described in Kibenge et al. [36]. All samples were screened for PRV targeting the L1 gene segment as described in Kibenge et al. [36]. In brief, Ct values ≤ 40 were considered positive.

### Statistical analyses

The data files used in the following analyses are available in S3 and S4 Tables.

The relationship between the viral screening results and exposure to salmon farms was first examined using a cluster analysis on the proportions of PRV-positive test results within farmed fish (Atlantic salmon and steelhead) and the nine wild fish regions (all species combined). Additionally, logistic regression analyses were used to: (a) probe for potential underlying causes for the geographic patterns in these proportions, (b) generate leads for further investigation, and (c) check for the potential that any apparent patterns could be attributable to other causes. The focus in the logistic regression analysis was on levels of exposure to salmon farms, return migration challenge, and host species. Lastly, the proportions of Atlantic salmon testing positive for PRV were assessed for inter-annual variation using likelihood-based inference.

Because so little is known about the potential epidemiological interactions between farmed and wild salmon in the North Pacific, an exploratory approach to our analyses was used. Thus, in keeping with the spirit of exploratory data analysis [43], we adopted a flexible approach to the selection of statistical methods and models, and put forward our conclusions as hypotheses worthy of further attention.

#### Cluster analysis

To perform the cluster analysis on the regional proportions of PRV results we applied the agglomerative, hierarchical clustering method based on cluster centroids as implemented in the SAS^®^ CLUSTER procedure, SAS software, Version 9.4. In keeping with commentary in SAS 2013, the centroid method was selected to avoid giving too much influence to the much larger proportion of PRV positive fish in the farmed Atlantic salmon category.

#### Logistic regression analysis

The logistic regression analysis was conducted solely on the wild fish. The factors of primary interest were: salmon farm exposure, migration challenge, and host species.

The number of categories was restricted to avoid the potential for over-parameterization. Farm exposure and migration challenge were categorized as low or high as described above. Host species were reduced to four taxonomic units among the wild fish by combining lineages that had not diverged prior to approximately 7.5 million years ago [44, 45]–chinook-coho salmon with 168 samples, chum-pink salmon with 175 samples, sockeye salmon with 220 samples, and rainbow-cutthroat trout, with 38 samples.

Two other factors, life stage and year, were included in the logistic regression analysis to probe for potential confounding effects. The wild salmon life stages were divided into 2 categories: juveniles (192 fish) and adults (409 fish).

Observations used in the logistic regression analysis were limited to 2012 and 2013, the years for which farmed and wild salmon were concurrently sampled in sufficient numbers. There was insufficient data to extend formal inferences to other years. There were too few degrees of freedom, and the standard assumption of independence between years that underlies the usual models for random effects would have been compromised if, for example fish returning at ages 4 and 5 from the same cohort were both exposed to the same PRV source at an earlier life stage. Furthermore, Taksdal [46] highlights the potential both for differences in virulence between virus subtypes, and for relatively abrupt changes in viral-subtype presences that could produce sudden jumps in the proportions of positive tests. Both of these events would reduce the comparability of years in which only farmed Atlantic or wild salmon were collected. Such complex behavior calls for more elaborate modelling. Hence, inferences have been limited to 2012–2013, year effects were treated as fixed, and Oweekeno Lake data was not included in the analysis.

Furthermore, there were sufficient numbers of observations to assess the main effects of each of the factors, but not necessarily for interactions between them (see S1 File for further explanation).

Finally, a random effect associated with the within-cluster correlation of fish obtained from the same location and year was included to account for potential dependency in PRV presence among fish sampled from the same effective host population. This term additionally compensates for cross-contamination within a sampling event, as this would have had a comparable impact to the contagious spread of virus within a school of fish before they were caught.

A more formal description of the statistical model is provided in S1 Text.

#### Model selection

We used a stepwise approach to our logistic regression (starting with a full model) to screen for potentially influential factors. To reduce the likelihood of deleting potentially important variables in this exploratory analysis, we planned to remove, at each deletion step, the variable with the highest *p*-value from the model only if its *p*-value exceeded 0.10. Competing methods based on AIC and other similar measures of goodness of fit were complicated by occasional cases of missing information on some variables. Hence, the stepwise approach was more appropriate, and generated a preferred model after only two deletion steps. All mixed-effects logistic regression inferences were performed using the SAS GLIMMIX procedure as implemented in SAS software, Version 9.4.

#### Comparative analysis of test results on farmed Atlantic salmon

We also formally compared the proportions of PRV-positive tests for the farmed Atlantic salmon between 2012 and 2013. Because multiple fish were purchased from the same outlet on the same day, we needed to account for potential dependence within such clusters of sampled fish generated by factors such as a common farm of origin and cross-contamination in processing and handling during harvest. We did so by incorporating a random effect term similar to that used in the logistic regression model. Details are provided in S2 Text.

## Results

**Fig 2:**
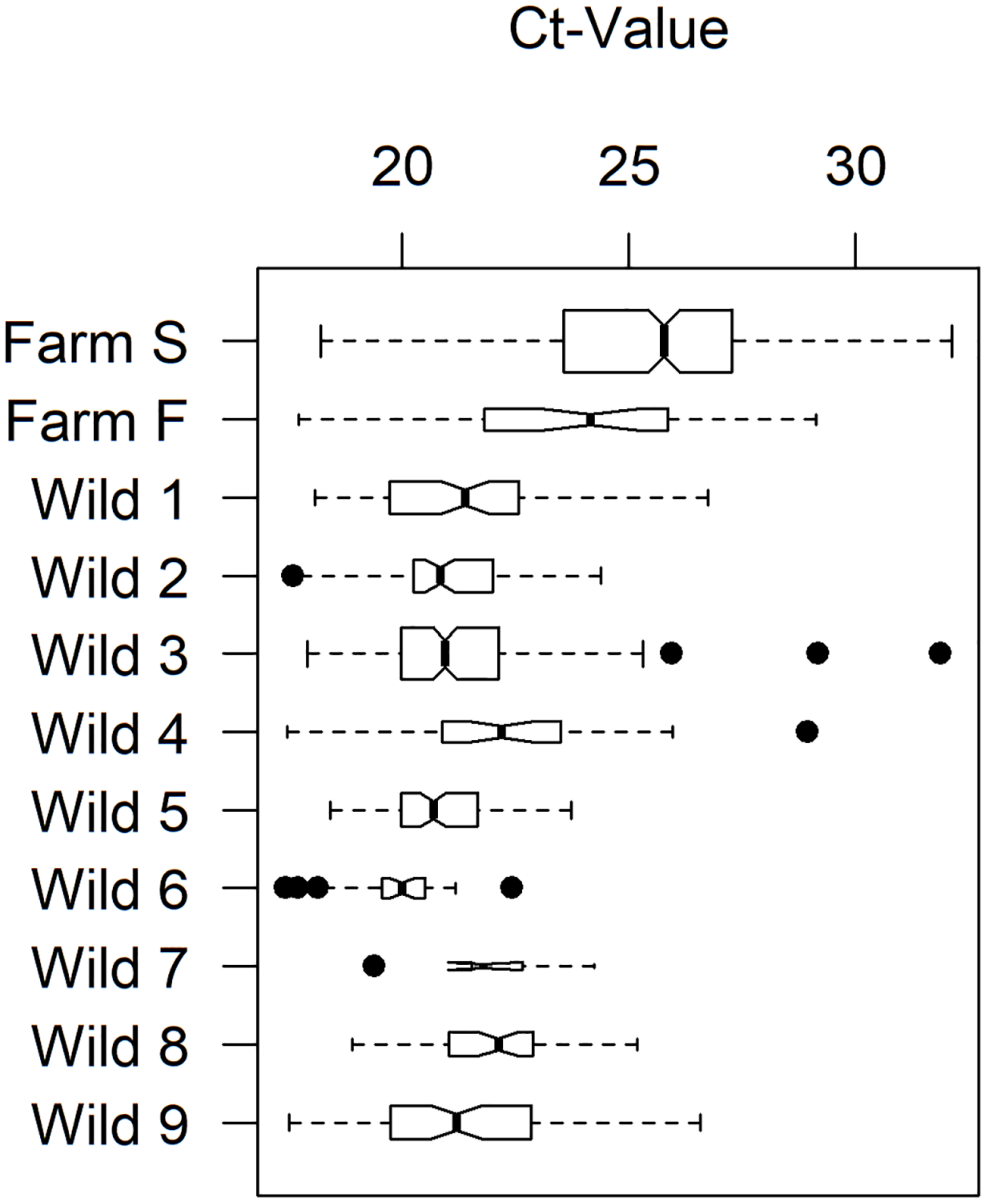
Internal control ELF-1α Ct values indicating sample quality. Ct values <30 are considered of sufficient quality for RTqPCR viral screening.

The farmed fish generated slightly higher ELF-1α Ct values, in keeping with the unavoidable delay in tissue preservation of market-sourced fish; however, tissue quality was suitable for RT-qPCR testing across all samples (Fig 2).

**Fig 3:**
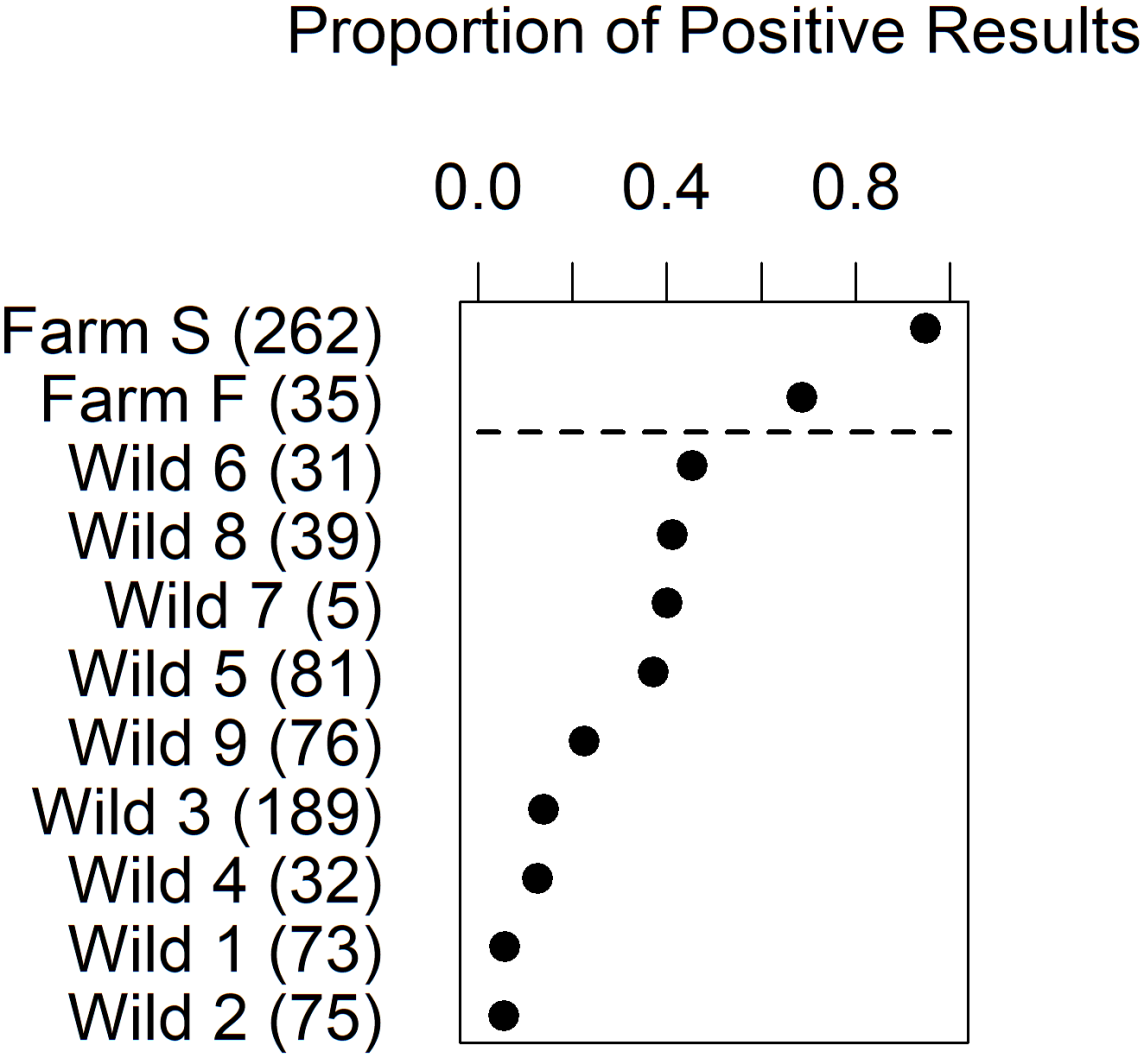
Proportions of PRV RT-qPCR-positive results. Results are arranged in decreasing order. The “Wild” designations reflect the Region numbers in Fig 1; i.e., Wild 1 is from Region 1. Numbers of fish sampled are provided in parentheses on the horizontal axis labels. Relevant estimates and confidence limits for key differences in this figure were generated by the logistic regression modelling where the effects of potential confounding variables could be filtered out (Fig 5).

Raw proportions of PRV-positive tests are shown in Fig 3. PRV infection was highest among the farmed salmon categories; Atlantic salmon (95%) and steelhead (69%). The highest proportions of PRV-infected wild salmonids were from the high exposure regions, *i*.*e*., Regions 5–8, including the lake with a steelhead farm and the highly exposed inshore archipelago environments (37–50%). The proportion of PRV infection declined between the highly exposed lower (41%) and upper (22%) Fraser River. The lowest proportions were in Regions 1 and 2, furthest from salmon farms (5%). In addition, Cultus Lake trout were highly infected with PRV (76%) (Lake c, Fig 1), while only 3% of the salmonids in Oweekeno Lake were infected with PRV (Lake a, Fig 1, S1 Table).

**Fig 4:**
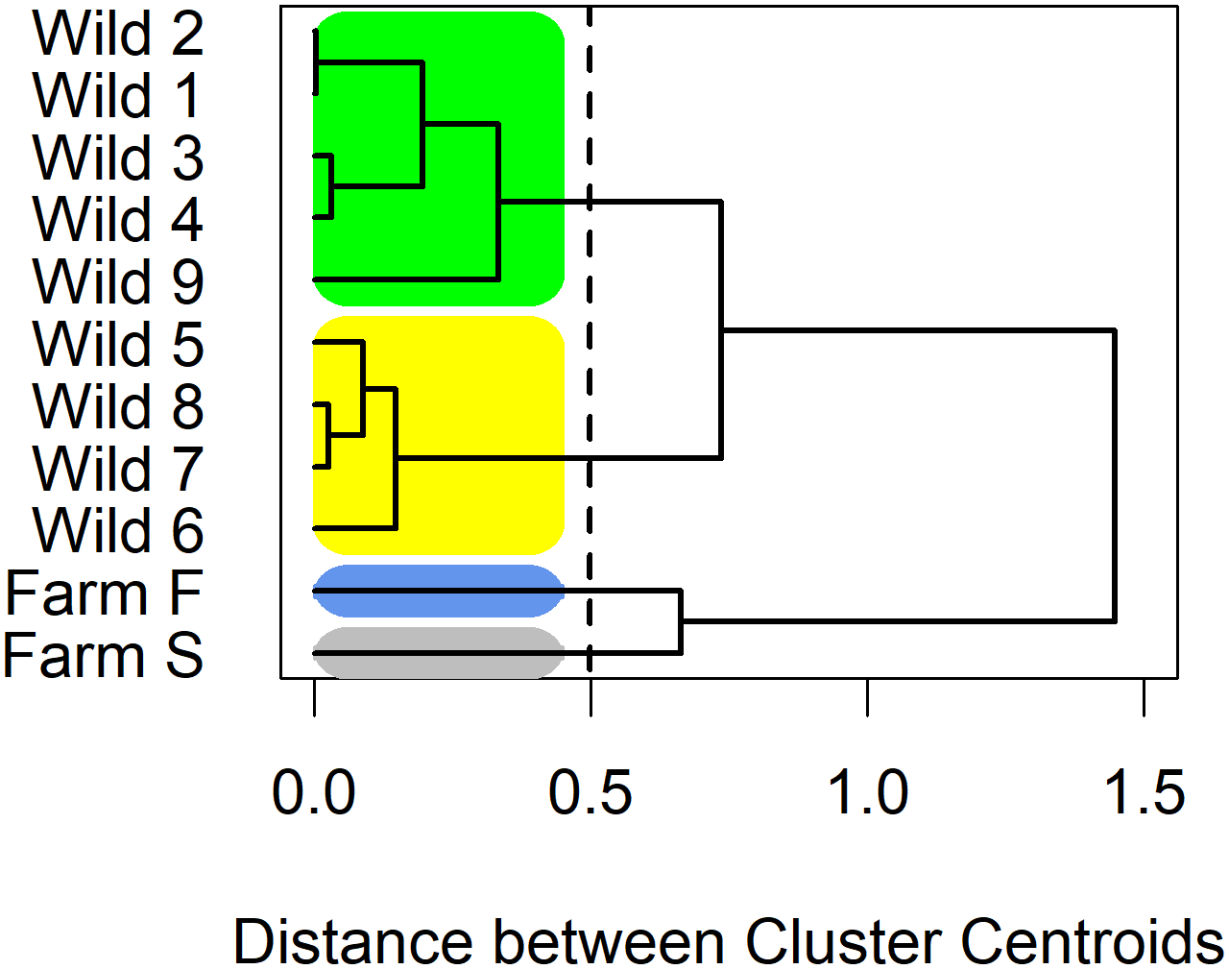
Hierarchical cluster analysis of test results by region and farmed categories.

A complementary perspective emerged from the cluster analysis on these proportions (Fig 4). The two farmed fish species each formed distinct, single-element clusters. All high exposure regions, except Region 9 (post high migration challenge) appear in the yellow cluster. The green, less homogeneous cluster includes the high migration challenge Region 9 with all the low exposure regions.

Details of the stepwise logistic regression procedure are summarized in S5 Table Summary of stepwise regression process. Two factors were dropped in the stepwise regression: “species group” and “life stage”, and then the algorithm terminated.

All of the fixed factors in the preferred model (year, farm exposure, and migration challenge) were significant (*p* < 0.01, Table 2).

**Table 2:** Summary of results for the preferred model. The SAS-generated table shows results of tests generated by dropping each factor from the model containing all three factors, with each factor replaced in the model before the next deletion. Degrees of freedom were calculated by SAS with a Satterthwaite correction. Estimates of the odds ratios were obtained by exponentiating the estimated coefficients for the log-odds ratios.

Type III Tests of Fixed Effects							
Effect	Coeff.	SE	Num. DF	Den. DF	*F*	*P*	Odds Ratio
**Year**	2.17	0.615	1	98.3	12.40	0.0007	8.72
**Exposed**	1.55	0.587	1	91.5	6.97	0.0097	6.97
**Challenged**	2.60	0.764	1	189.8	11.57	0.0008	13.44

**Fig 5:**
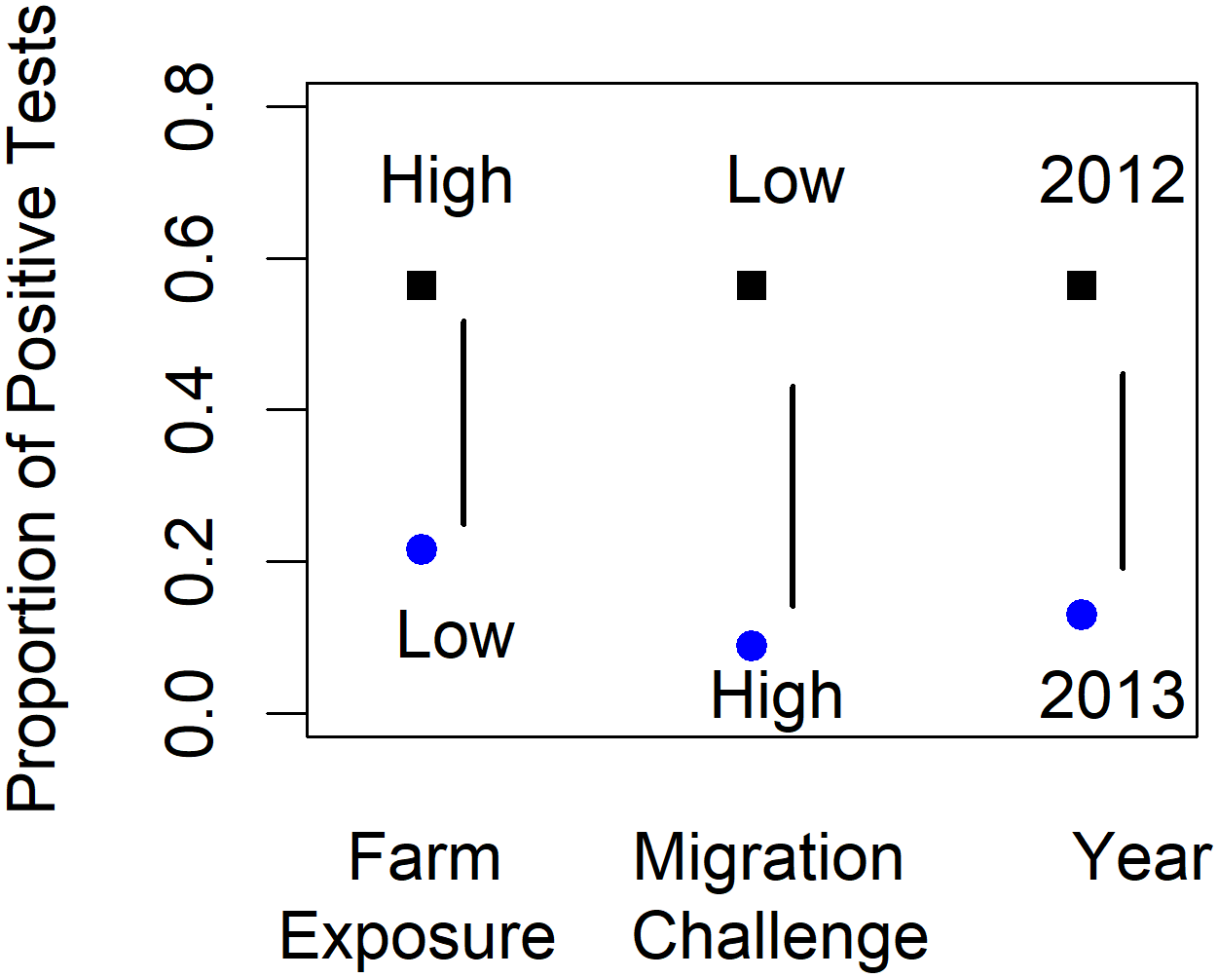
Least-squares mean proportions for RT-qPCR positive test results. The black squares provide a reference estimate for fish at high farm exposure level, and low migration challenge level in 2012 (the ‘common reference’). The blue circles are least-squares mean proportions with each of these factors switched in turn to the opposite level, with the other factors left at the common reference level. The vertical bars cover approximately 2 standard errors. Where a vertical bar does not span the gap between the two estimates, the difference is significant at approximately the 5% level in a test against a two-sided alternative.

The proportion of PRV-infection in both wild and farmed salmon declined substantially between 2012 and 2013. For highly exposed wild salmon that had not faced a major migration challenge, the least-squares mean estimate of this proportion declined from 0.564 in 2012 to 0.129 in 2013 (Fig 5). The corresponding decline for farmed Atlantic salmon, from 0.974 to 0.790, was also strongly significant (*p* = 0.002 from the modelling procedure outlined in S2 Text).

In addition to year, the effects of the other two factors, exposure and migration challenge, were also estimated to be large, though with substantial standard errors (Fig 5). The estimated effect of the most significant of all three factors, migration challenge, was also the largest. Fig 5 shows that, for high-exposure wild salmon in 2012, there was over a six-fold decline in the estimated proportion of PRV-positive test results from (a) fish in the low-challenge category to (b) those in the high-challenge category. This estimated decline is commensurate with the observed declines (i) between Regions 8 and 9 (the lower and upper Fraser River areas) and (ii) between the lower and higher elevations in Region 1 in northern BC (Table 3). Fig 5 also shows that, for low-challenge wild salmon in 2012, there was over a two-fold decline in the estimated proportion of PRV-positive test results from (a) fish in the high-exposure category to (b) those in the low-exposure category.

**Table 3:** Observed proportions of positive PRV tests by migration challenge level for regions with substantial numbers of migration-challenged fish (low exposure region 1 and high exposure regions 8 & 9). Numbers in brackets reflect numbers of positive tests per fish sampled.

	Migration Challenge	
Region(s)	Low	High
**1**	0.429 (3/7)	0.018 (1/56)
**8 & 9**	0.410 (16/39)	0.224 (17/76)

## Discussion

The results of this work suggest that exposure to salmon farms has a strong association with increased risk of PRV infection in wild salmonids, and that the proportion of PRV-infected wild vs. farmed salmon can vary synchronously between years. In addition, the decline in PRV infection between the low and high migration challenge groups suggests that PRV infection may reduce a host’s capacity to complete a challenging upriver migration, thereby reducing reproductive fitness. We stress the correlational nature of the present findings, but believe, in keeping with the Precautionary Principle, that they warrant further research attention due to the high ecological, economic, and cultural value of wild Pacific salmon.

The hierarchical cluster analysis of PRV-infection proportions showed a clear separation between the more highly infected farmed Atlantic salmon and all categories of wild Pacific salmon (Fig 4). This demonstrates the significantly greater potential infection pressure imposed by farmed salmon in comparison to wild salmon. Further, the logistic regression analysis has demonstrated that higher exposure to farmed salmon is associated with a significant increase in the proportion of PRV infected wild salmon. This is a plausible result as 79–95% of farmed salmon tested positive for PRV and wild fish in the regions where salmon farms operate among retentive currents would likely experience a higher contact rate with infectious PRV particles than wild salmon elsewhere. This result is in keeping with other research findings [6,47]. Deterministic modeling of water-borne infectious particles demonstrates that a high number of shedding hosts elevates the localized concentration of infectious particles thereby increasing the rate of infection in susceptible hosts [47]. This model is well supported by the empirical evidence that farmed salmon epizootics tend to cluster in both space and time (as reviewed in [6]). PRV has also been shown to be highly infectious both among and between species with transmission occurring from Atlantic salmon to both Atlantic and Pacific salmon through experimental cohabitation challenges [9, 22, 23, 24, 34]. While the exact mechanism of PRV transmission remains unknown, Hauge et al. [48] show that faecal virus shedding may release a significant amount of infectious particles into the water. While the heightened proportions of PRV-infection in wild salmon from high exposure regions provides some epidemiological evidence of PRV transmission between farmed and wild fish, the presence of PRV in low exposure populations suggests that transmission may also occur between individuals of wild populations in the open Pacific, though perhaps at a lower frequency. Together, these findings raise the concern that point-source pathogen release from aquaculture facilities may affect both populations directly exposed, and those that are not directly exposed to salmon farms. Siah et al. [33] also suggested that wild-to-wild transmission best explains the homogenous distribution of PRV S1 sequence types in the eastern Pacific.

By contrast in Norway, Garseth et al. [5] proposed that PRV transmission between low density wild Atlantic salmon during their at sea phase likely plays a minor role in infection rates. However, it is possible that the more abundant wild salmon populations in the northeastern Pacific may provide better opportunities for PRV transmission. Our data provides some evidence for PRV transmission between wild fish, as low exposure populations also carry PRV. However, the higher PRV infection rates among those wild salmon in closer contact with Atlantic salmon also provides provisional evidence of PRV transmission in at least one direction between wild and farmed salmon. Additionally, the significant effect of year on the PRV infected proportion, which acts in the same direction for both wild and farmed salmon, also appears to corroborate the hypothesis that PRV prevalence in wild salmon is epidemiologically linked to prevalence in farmed Atlantic salmon. Garver et al.’s [23] findings that PRV can be transmitted from Atlantic salmon to Pacific salmon but not vice versa provides support for a dominant farmed-to-wild transmission route.

Additionally, this study demonstrates strong evidence generated collectively from two regions in BC of a negative association between increased migratory challenge and PRV-positive proportions in return-migrating wild adult salmon. Fewer infected adults of any species were detected at higher vs. lower elevations in the Fraser River, as well as tributaries of the Skeena and Nass rivers in northern BC. This association points to a cost of infection from PRV to the fitness of wild Pacific salmon. While the pathogenicity of PRV in wild Pacific salmon has been questioned (e.g., [26, 32]), PRV-associated disease states (i.e., HSMI [13] and Jaundice Syndrome [16, 17]) are characterized by lethargy and erratic swimming behaviour [12], which would have more serious consequences for wild Pacific salmon than for farmed Atlantic salmon in net pens. The statistical modelling performed accounted for potential confounding effects from year, exposure level, salmonid host species, and life stage, and still found strong evidence of a decline in the infected proportion of salmon at higher elevations. However, it is possible that some other factor not included in the model could account for this change in proportions. Further investigations employing the tracking of the in-river fates of individual salmon by biotelemetry, as has been demonstrated by Jeffries et al. [49], and Miller et al. [37, 50], can better resolve confounding variation possibly associated with the migration timing of specific stocks and the timing of sampling events. However, with the geographic scale and numbers of fish used in the present study, it was infeasible to employ such technologies. Nonetheless, the evidence of lower PRV presence in salmon at higher elevations has important potential implications regarding fitness costs and population impacts of PRV on wild salmon. Similar findings were also reported by Miller et al. [37], who found PRV infection to be significantly associated with en-route migration losses for Chilko Lake sockeye salmon, which are challenged by an arduous 1,172m elevation gain in their return migration (Region 9, Lake b, Fig 1). In contrast, these authors reported that PRV infection was not significantly associated with migration losses into the lower elevation Shuswap Lake watershed (elevation 350m).

The PRV infections detected in salmonids in low-lying lakes, Cultus (elevation 47 m) and Oweekeno (elevation 15 m), and in particular the higher proportion of positives in Cultus Lake trout where anadromous salmon entering the lake have been highly exposed to farmed salmon potentially on both seaward and return migrations, provide a contrast to the observed reduction in PRV in fish sampled at higher elevations. This contrast suggests the following hypotheses for future research: (i) PRV-infected wild fish are less able to meet the challenge of migrating into higher elevations above sea level, (ii) easily accessed, low-lying lakes lack the infection filtering effects of return migrations with greater challenges and may be more vulnerable to the introduction of aquaculture-source viruses via infected anadromous salmonids than high elevation habitat, and (iii) resident trout or other fish species in these lakes may act as viral reservoirs increasing the complexity of PRV transmission dynamics and potentially exposing successive generations of salmonids to infection.

PRV has previously been shown to have a broad host range among salmonids in the Pacific and Atlantic [this study, 5, 23, 26, 32, 33, 36], including a first report in this study of a positive test for Dolly Varden char (from Oweekeno Lake, Fig 1, Lake a, S1 Table). Positive PRV results have been reported for some non-salmonid marine fish in coastal Norway as well [51]. The consequences of these potentially complex host-pathogen dynamics for sustaining infections in wild salmon populations are unknown, but their prospect raises important questions regarding the vulnerability of low-elevation salmonid populations to viral disease. Future work should attempt to identify competent host species, and to characterize viral reservoirs in addition to Atlantic salmon farms, particularly in light of the collapse of both the low-lying Cultus and Oweekeno Lake sockeye salmon populations to less than 1% of their historic spawner returns with no clear cause, despite significant restoration efforts [52, 53].

Recent research on PRV points to mechanisms through which the virus might impact the capacity of a salmon to complete a challenging migration to reach its spawning grounds [13, 22]. PRV has been found to proliferate in the erythrocytes, with possible implications for oxygen transport and swimming performance [22]. Research on PRV infection in Atlantic salmon hosts has also shown that PRV has a transient acute infection stage during which innate antiviral pathways are strongly upregulated [9]. Activation of these immune system pathways has been shown to have both direct and indirect energetic costs to a host [54]. While a similar level of immune activation in response to PRV infection has not been shown for Pacific salmon species [24], this could have other explanations beyond a total lack of pathogenicity, specifically: differences in pathogenicity among described and uncharacterized PRV strains, host species/virus strain interactions, and inferential complications arising from the current inability to culture PRV in fish cell lines.

Histopathological examination of samples has value in confirming disease state and reinforcing the association between a condition and any impact to fitness; however, this approach was not employed in the present study as it is considered unlikely that wild salmon will progress to clinical disease before being targeted by predation [55]. A potentially more profitable approach employed by Miller et al. [55] uses modern molecular methods to predict the pathogenic outcomes of infection for salmon at early stages of infection. These authors have found that gene expression biomarkers for active virus infection can differentiate between both Atlantic salmon with HSMI and Pacific salmon species with Jaundice Syndrome (also strongly associated with PRV [16, 17]) from virus-negative fish and from fish with clinical diseases caused by other pathogen types [55]. It is hoped that greater numbers of future studies will take this approach in order to strengthen or refute the associations found herein, and more fully understand the consequences of viral pathogens like PRV for the fate of infected wild salmonids.

## Conclusions

This study provides the first evidence that (i) exposure to farmed Atlantic salmon is associated with infection of wild Pacific salmon with PRV, a virus of significant concern to both the aquaculture industry and wild fisheries management, and (ii) that PRV infection may impair the capacity of wild salmon to complete a challenging spawning migration, with the potential for population-level impacts. The evidence, based solely on molecular screening tests from this observational study, and constrained by limited access to farmed Atlantic salmon samples of known provenance, cannot be definitive. Nonetheless, we view it as providing an early warning sign of a potentially serious problem that warrants immediate and ongoing research. Research into the fitness impacts to wild Pacific salmonids of farmed salmon pathogens is needed in wild fish populations in addition to controlled laboratory environments, and could provide valuable insights useful for the management of critically declining wild salmon populations.

## Supporting information

S1 Table Oweekeno Lake salmonid samples. (DOCX)

S2 Table Oweekeno Lake data file. (XLSX)

S3 Table Wild salmonid data file. (XLSX)

S4 Table Farmed fish data file. (XLSX)

S5 Table Summary of stepwise regression process. (DOCX)

S1 File Numbers of 2012–3 sampled salmonids by species group and migration challenge categories. (DOCX)

S1 Text Logistic regression model summary. (DOCX)

S2 Text Technical details of comparative analysis on farmed Atlantic salmon. (DOCX)
