# Correction: The effect of exposure to farmed salmon on piscine orthoreovirus infection and fitness in wild Pacific salmon in British Columbia, Canada

**DOI:** 10.1371/journal.pone.0248912

**Published:** 2021-03-16

**Authors:** Alexandra Morton, Richard Routledge, Stacey Hrushowy, Molly Kibenge, Frederick Kibenge

The supplementary datafiles for this paper did not include Ct values for the PRV tests. We now provide these values in S1 File and S2 File of this Correction.

The performance of the real-time RT-PCR assay for PRV was based on published protocols (the assay used primers and probe by Haugland et al., 2011, Journal of Virology 85:5275–5286, and reaction conditions by Palacios et al., 2010. PLoS ONE 5:e11487). A cut-off Ct value of 40 was used as established by Løvoll et al., 2012. Ct values ≤ 40 were considered positive.

The Ct values in this study were automatically reported by the real-time instrument (LC 480, Roche), which reported Ct values above 40 as 0. Thus, any samples generating Ct values that were > 40 were designated as negative along with samples for which no Ct value was generated.

Minor transcription errors in the file ‘S3_Table Wild Fish Data" have been corrected in the revised version of this file attached to this correction notice. We make the following minor corrections: (i) The percentage of positive tests in the farmed Atlantic salmon sampled fish should be lowered from 95% to 93%, and (ii) the percentage of positive tests in the farmed steelhead sampled fish should be raised from 69% to 71%. These minor corrections had no impact on the results of the statistical analyses reported in the paper or the subsequent discussion or conclusions.

## Supporting information

S1 File Amended version of the Wild salmonid data file (S3 Table in [1]). (XLSX)

S2 File Amended version of the farmed fish data file (S4 Table in [1]). (XLSX)
