# Correction: The effect of exposure to farmed salmon on piscine orthoreovirus infection and fitness in wild Pacific salmon in British Columbia, Canada

**DOI:** 10.1371/journal.pone.0282687

**Published:** 2023-03-02

**Authors:** Alexandra Morton, Richard Routledge, Stacey Hrushowy, Molly Kibenge, Frederick Kibenge

Following publication of [1], an amended version of S3 Table in [1] was published in a Correction [2].

There are errors in S1 File in [2]. Specifically,

1. A PRV Ct value is missing in cell H29
2. A pivot table was erroneously included that is not part of the underlying data for the published study [1]

Here, the authors provide a corrected version of S3 Table of [1] (S1 File) that includes the amendments described in [2] and addresses the issues above by providing the missing PRV Ct value and removing the pivot table. As such, the S1 File of [2] is superseded by the S1 File provided in this notice.

## Supporting information

S1 File Amended version of the Wild salmonid data file (S3 Table in [1]). (XLSX)

## References

[1] Morton A, Routledge R, Hrushowy S, Kibenge M, Kibenge F (2017) The effect of exposure to farmed salmon on piscine orthoreovirus infection and fitness in wild Pacific salmon in British Columbia, Canada. PLoS ONE 12(12): e0188793. 10.1371/journal.pone.0188793 29236731 PMC5728458

[2] Morton A, Routledge R, Hrushowy S, Kibenge M, Kibenge F (2021) Correction: The effect of exposure to farmed salmon on piscine orthoreovirus infection and fitness in wild Pacific salmon in British Columbia, Canada. PLoS ONE 16(3): e0248912. 10.1371/journal.pone.0248912 33725019 PMC7963067

