# Supplementary material for: The effect of exposure to farmed salmon on piscine orthoreovirus infection and fitness in wild Pacific salmon in British Columbia, Canada
Source: PLoS One. 2017 Dec 13;12(12):e0188793. doi: 10.1371/journal.pone.0188793 (PMC5728458; doi:10.1371/journal.pone.0188793)
Supplement: S1 Table — (DOCX) [file pone.0188793.s001.docx]

**S1 Table.** **Oweekeno Lake salmonid samples.**

Juvenile Sockeye salmon from Oweekeno include both freshwater fry and saltwater smolt stages.

| **Species** | **Number screened** | **Number positive** | **Percent (%) PRV positive** |
| --- | --- | --- | --- |
| Sockeye*  Adults  Juveniles  Total | 74  270  344 | 2  6  8 | 2.7%  2.2%  2.3% |
| Chinook | 41 | 1 | 2.4% |
| Cutthroat trout | 19 | 2 | 10.5% |
| Dolly Varden (*Salvelinus malma*) | 10 | 1 | 10.0% |

*All salmonids other than sockeye salmon were > 1 year old.
