# Supplementary material for: The effect of exposure to farmed salmon on piscine orthoreovirus infection and fitness in wild Pacific salmon in British Columbia, Canada
Source: PLoS One. 2017 Dec 13;12(12):e0188793. doi: 10.1371/journal.pone.0188793 (PMC5728458; doi:10.1371/journal.pone.0188793)
Supplement: S5 Table — (DOCX) [file pone.0188793.s005.docx]

**S5 Table. *P*-values for variables in the stepwise regression with the selected model highlighted in italics.** Each row in the table corresponds to a step in the analysis. The first line shows the fit of the initial (full) model. The variable with the largest *p*-value (Species Group) was then dropped from this model. The analysis terminated after five such steps.

| **Step: With Decision for Next Step** | **Year** | **Species Group**  **(SpGp)** | **Life**  **Stage**  **(LfSt)** | **Farm Exposure Status** | **Migration Challenge Status** |
| --- | --- | --- | --- | --- | --- |
| **1: Drop SpGp** | 0.0004 | 0.4621 | 0.2103 | 0.1420 | 0.0037 |
| **2: Drop LfSt** | 0.0006 | NA | 0.3742 | 0.0543 | 0.0026 |
| **3: Re-add SpGp** | *0.0007* | *NA* | *NA* | *0.0097* | *0.0008* |
| **4:Re-drop SpGp** | 0.0005 | 0.6355 | NA | 0.0187 | 0.0013 |
| ***5: Stop*** | *0.0007* | *NA* | *NA* | *0.0097* | *0.0008* |
