# Supplementary material for: The effect of exposure to farmed salmon on piscine orthoreovirus infection and fitness in wild Pacific salmon in British Columbia, Canada
Source: PLoS One. 2017 Dec 13;12(12):e0188793. doi: 10.1371/journal.pone.0188793 (PMC5728458; doi:10.1371/journal.pone.0188793)
Supplement: S1 File — (DOCX) [file pone.0188793.s006.docx]

**S1 File.** Numbers of sampled fish included in the model by all combinations of the factors delineating species category and the degree of challenge experienced in any return migration route. (Note that the overall total is only 591 because there were 10 sampled fish for which, due to incomplete records, the migration challenge status could not be assessed.)

| **Species** | **Migration Challenge** | | **Total** |
| --- | --- | --- | --- |
| **Category** | **Low** | **High** |  |
| **chinook-coho** | 147 | 19 | 166 |
| **pink-chum** | 164 | 6 | 170 |
| **sockeye** | 121 | 96 | 217 |
| **trout** | 26 | 12 | 38 |
| **Total** | 458 | 133 | 591 |

There were sufficient numbers of observations to assess the main effects of each of the factors, but not necessarily for the interaction effects between them. For example, consider farm exposure and migration challenge (Table 2). With only 6 fish in the pink-chum/high migration challenge cell, the proportion for this cell was not generated with sufficient precision. Because interaction effects depend on such estimates, results of interactions between factors (or any others) in our statistical model.
