# Supplementary material for: The effect of exposure to farmed salmon on piscine orthoreovirus infection and fitness in wild Pacific salmon in British Columbia, Canada
Source: PLoS One. 2017 Dec 13;12(12):e0188793. doi: 10.1371/journal.pone.0188793 (PMC5728458; doi:10.1371/journal.pone.0188793)
Supplement: S1 Text — (DOCX) [file pone.0188793.s007.docx]

**S1 Text Logistic regression model summary**

The model stipulates that the proportion (*p*) of positive PRV test results within each source-year cluster of sampled fish is generated independently within each cluster according to the following:

*p* = *f*(a linear combination of main effects of fixed factors plus a random intercept)

+ a binomially generated random error term,

where *f* is the standard logistic function,

*f*(*x*) = e*^x^*/(1+ e*^x^*), and

the random intercept term is a normally distributed with the same mean and variance for each cluster.
