# Supplementary material for: The effect of exposure to farmed salmon on piscine orthoreovirus infection and fitness in wild Pacific salmon in British Columbia, Canada
Source: PLoS One. 2017 Dec 13;12(12):e0188793. doi: 10.1371/journal.pone.0188793 (PMC5728458; doi:10.1371/journal.pone.0188793)
Supplement: S2 Text — (DOCX) [file pone.0188793.s008.docx]

**S2 Text. Technical details of comparative analysis on farmed Atlantic salmon**

To formally compare the proportions of PRV-positive tests for the farmed Atlantic salmon between 2012 and 2013, we used a generalized binomial, likelihood-based approach. Because multiple fish were purchased from the same outlet on the same day, we needed to account for potential dependence within such clusters of sampled fish generated by factors such as a common farm of origin and cross-contamination in processing and handling. We did so by incorporating a random effect term as follows: Within each cluster, the number of positive PRV test results was assumed to have a binomial distribution, conditional upon the value of the binomial probability parameter for that cluster. This parameter was in turn assumed to vary between clusters according to a beta distribution. This parameter, which had a common value for fish within any given cluster, generated the needed within-cluster correlation. We then used maximum likelihood estimation to estimate the parameters and test the null hypothesis that there was no difference between years. The analysis was performed through a custom-written program in the statistical package, R, with the maximization performed by the R function, ‘optim’, using method, 'L-BFGS-B', a quasi-Newton method modified for handling bounds on the parameters.
